# circHIPK3 Acts as Competing Endogenous RNA and Promotes Non-Small-Cell Lung Cancer Progression through the miR-107/BDNF Signaling Pathway

**DOI:** 10.1155/2020/6075902

**Published:** 2020-09-14

**Authors:** Weijun Hong, Yajuan Zhang, Jingyi Ding, Qilian Yang, Haixiang Xie, Xiwen Gao

**Affiliations:** ^1^Department of Respiratory Medicine, Minhang Hospital, Fudan University, China; ^2^Pharmacy Department, Minhang Hospital, Fudan University, China; ^3^Department of Emergency Medicine, Minhang Hospital, Fudan University, China

## Abstract

Circular RNAs (circRNAs) act as a crucial part in many human diseases, particularly in cancers. circRNA HIPK3 (circHIPK3) is a special circRNA that may participate in the oncogenesis of non-small-cell lung cancer (NSCLC), even though its latent regulatory mechanism is not very clear. Here, we studied the roles of circHIPK3 in NSCLC. qRT-PCR assay was applied to study the expression of circHIPK3 in NSCLC. The influence of circHIPK3 on NSCLC was estimated by silencing circHIPK3 and miR-107 mock transfection and brain-derived neurotrophic factor (BDNF) overexpression, and the correlation between circHIPK3, miR-107, and BDNF was evaluated by dual-luciferase reporter assay. The results showed that circHIPK3 expression was upregulated in NSCLC cells. circHIPK3 knockdown inhibited the migration and proliferation of NSCLC cells by promoting the expression of miR-107. circHIPK3 could be used as a miR-107 sponge to promote BDNF cell proliferation. The dual-luciferase reporter assay proved that miR-107 was the target of circHIPK3, and miR-107 had an interaction with the 3′untranslated region of BDNF. miR-107 overexpression inhibited BDNF-mediated NSCLC cell proliferation. These results indicate that circHIPK3 promotes tumor progression through a new circHIPK3/miR-107/BDNF axis, which offers potential markers and medical treatment for NSCLC.

## 1. Background

Cancer-related diseases have become the biggest leading cause of death worldwide. Lung tumor ranked the first site of all cancers and accounted for 11.6 percent of newly diagnosed cancers and 18.4 percent of cancer-related deaths from the latest report in 2018 [1, 2]. Lung cancer (LC) includes two main types: small-cell lung cancer (SCLC) and non-small-cell lung cancer (NSCLC), which account for about 85% of all LC cases [3]. As imaging modality in medical diagnosis is widely used, the accuracy of diagnosis in NSCLC has been greatly improved [4]. Early diagnosis of patient with NSCLC has not yet been satisfactorily effective for expensive examination fees and low accuracy of clinical image interpretation. The five-year survival rate of patient with NSCLC is often low, due to the diagnosed time in an advanced stage and the lack of tumor-specific agents [5]. Therefore, it is important to find an appropriate target of NSCLC.

circRNAs are a class of RNA modules that form a continuous cycle of covalent closures. Due to the limitations of technique and cognition, circRNAs are initially thought to be metabolic wastes of RNA missplicing. In recent years, circRNAs have been found to have many functions in the metabolic activities of cells. Unlike linear RNAs, circRNAs are relatively stable presenting in the body, because they have a unique ring-like structure [6–8]. The most common function of circRNAs is to act as miRNA sponges in the regulation of the cell signaling pathway. For example, Cao et al. found that hsa_circ_0037251 enhanced the progression of glioma via sponging miR-1229-3p [9]. Moreover, circRNAs can also engage in other cell activities. For instance, circRNA circ-Foxo3 can interact with RNA-binding proteins CDK2 and p21, leading to cell cycle arrest [10]. circRNAs can also strengthen the stability of mRNA by binding it, regulate parental gene transcription, and even translate into protein [8]. Previous studies have revealed the abnormal expression of circRNAs is associated with the development of cancer [11]. For example, Han et al. discovered circ-ABCB10 was remarkably upregulated in breast cancer tissue, promoting cell proliferation [12]. A recent study demonstrated that circ-ITCH was suppressed in bladder cancer samples, and upregulation of circ-ITCH inhibited bladder cell proliferation, migration, invasion, and metastasis [13]. There have been related researches about circHIPK3 in lung cancer. The result shows that the circHIPK3 can affect the related pathway through different regulatory axes [14, 15].

MicroRNA (miRNA) is also a very important class of noncoding RNA. It can regulate translation via interacting with the 3′UTR region of mRNA [2]. miRNAs play pivotal roles in the process of gene translation, acting as gene regulators, but miRNAs also are regulated by other factors, such as lncRNA and circRNA. For instance, it is demonstrated that lncRNA APF affects ATG7 expression by regulating miR-188-3p, thereby promoting autophagy death and myocardial infarction [16]. circRNA_010567 affects the expression of the miR-141 and TGF-*β*1 and promotes the resection of fibrosis-associated protein in cardiac fibroblasts [17]. Few studies have pointed out that the circRNAs serve as miRNA sponges in regulating the gene expression [18].

In this research, we found that circHIPK3 was strongly overexpressed in NSCLC tissue. Based on the finding, we conducted a series of assays to explore the roles of circHIPK3 in the progression of NSCLC. The results revealed that circHIPK3 could act as a sponge for miR-107 to promote the NSCLC cell tumorigenesis and relieve miRNA repression for downstream target gene BDNF. In short, our results showed that circHIPK3 might act as an oncogenic gene in NSCLC progression and could be a potential biomarker for screening and treatment of NSCLC.

## 2. Materials and Methods

### 2.1. Cell Culture

H1299, A549, and BEAS-2B were obtained from ATCC and cultured in DMEM (Life Technologies, Carlsbad, CA) with 10% fetal bovine serum (FBS), 100 U/mL penicillin, and 100 *μ*g/mL streptomycin, in a 37°C incubator containing 5% CO_2_.

### 2.2. Plasmid Construction and Transfection Assay

To overexpress miR-107, miR-107 mimics and miR-NC mimics were obtained from the GenePharma company. We used Lipofectamine 2000 (Invitrogen, CA) to transfect the cells with 50 nm mimics. To inhibit the expression of miR-107, NSCLC cells were transfected with miR-107 specific inhibitor (Invitrogen) for 48 hours prior to other experiments. For the expression analysis of BDNF, we used the PCDNA3.1 vector and transfected the plasmids into cells with Lipofectamine 2000. To analyze the expression of circHIPK3, Lipofectamine 2000 was used to transfect small interfering RNA (siRNA) for circHIPK3 (GenePharma) into H1299 and A549 cells at 50 nM.

### 2.3. Total RNA Isolation and Real-Time Fluorescent Quantitative PCR (qRT-PCR)

We extracted the total RNA by using TRIzol reagent (Invitrogen) and decided the concentration via the NanoDrop ND-1000. The primers for the detection of circHIPK3, miR-107, and BDNF were designed and purchased from GenePharma. qRT-PCR was applied using an AB7300 thermo-recycler (Applied Biosystems, Carlsbad, CA) with a TaqMan Universal PCR Master Mix. The relative expression of targets was determined by the 2^−*ΔΔ*Ct^ method.

### 2.4. Assay of Cell Proliferation

Cell growth ability was measured by using the CCK-8 kit depending on the instruction (Dojindo Laboratories, Japan) [19].

### 2.5. Transwell Assay

Transwell chambers (Corning, NY) were applied to observe the cell migration and invasion. After cultured for 2 days, the cells on the upper surface were removed. The cells on the lower surface were fixed and stained with DAPI. For the detection of invasion, the cells were seeded into the upper chamber which was precoated with a 2 mg/mL matrix gel.

### 2.6. Dual-Luciferase Reporter Assay

First, the mutant and wild-type circHIPK3 and BDNF1 3′UTR were cloned into pmirGLO vectors. WT/Mut-pmirGLO-circHIPK3 or WT/Mut-BDNF and miR-107 were cotransfected into NSCLC cells with Lipofectamine 2000. After transfection for 2 days, the cells were collected and detected the luciferase activity with the luciferase reporter assay system (Promega, Madison, WI). The relative activity was set as an internal control index of renal dual-luciferase.

### 2.7. Statistical Analysis

We used the SPSS software to analyze data. Student's *t*-test or the Mann-Wintney nonparametric test was used to determine the difference between two groups, and *p* < 0.05 was considered to be significant. The *p* value was corrected by the FDR (False Discovery Rate).

## 3. Results

### 3.1. circHIPK3 Downregulation Inhibited NSCLC Cell Proliferation and Invasion

**Figure 1:**
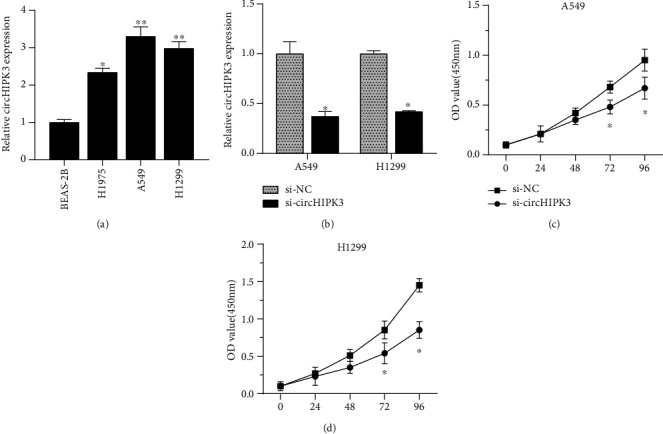
circHIPK3 downregulation inhibited NSCLC cell proliferation. (a) The circHIPK3 in the NSCLC cell lines was increased compared to that in BEAS-2B. (b) Effect of small interfering RNA (si-circHIPK3) directed against circHIPK3 in H1299 and A549 cells. (c, d) circHIPK3 silencing had an inhibitory effect on the proliferation of H1299 and A549 cells. ^∗^*p* < 0.05, ^∗∗^*p* < 0.01, and ^∗∗∗^*p* < 0.001.

**Figure 2:**
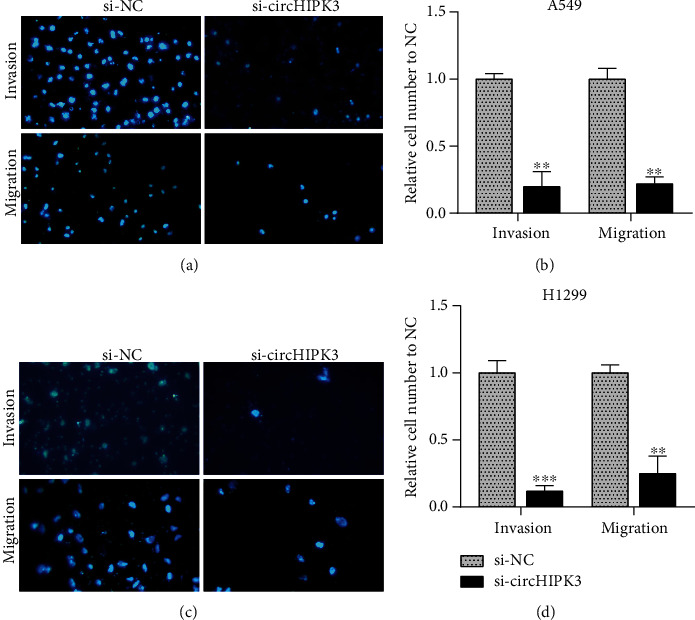
circHIPK3 downregulation inhibited NSCLC cell metastasis. (a, c) Picture of A549 (a) and H1299 (b) cells transfected with si-circHIPK3 and si-NC under a fluorescence microscope. (b, d) Knockdown of circHIPK3 suppressed cell metastasis in A549 (b) and H1299 (c). ^∗^*p* < 0.05, ^∗∗^*p* < 0.01, and ^∗∗∗^*p* < 0.001.

The potential regulatory mechanism of circHIPK3 was still unclear. The results showed that the circHIPK3 in the H1975, A549, and H1299 cell lines was increased compared to that in BEAS-2B (Figure 1(a)). H1299 and A549 cells were used for functional exploration, because their expression levels were the highest. To detect the potential function of circHIPK3 in the invasion and proliferation of NSCLC cells, siRNA cells were transfected for 48 h and compared with the NC groups. As presented in Figure 1(b), it was remarkable that the expression of circHIPK3 was downregulated. The CCK-8 assay showed that circHIPK3 silencing inhibited the proliferation of A549 and H1299 cells depending on the time (Figures 1(c) and 1(d)). Transwell assay also showed that silencing circHIPK3 had an inhibitory effect on the migration and invasion of NSCLC (Figures 2(a)–2(d)).

### 3.2. miR-107 Was a Target for circHIPK3 in NSCLC Cells

**Figure 3:**
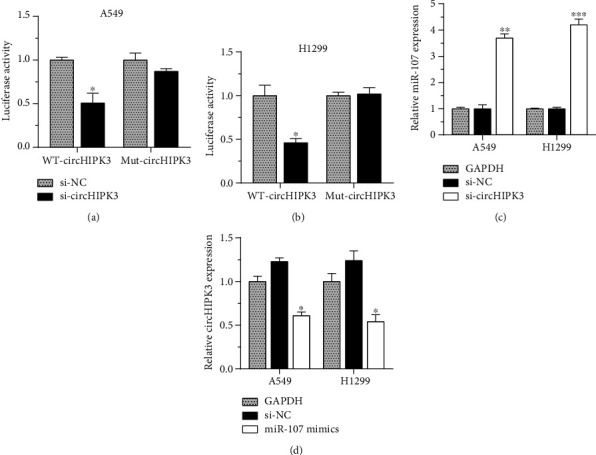
miR-107 was a target for circHIPK3 in NSCLC cells. (a, b) A549 (a) and H1299 (b) cells cotransfected with miR-107 and WT-circHIPK3 have lower relative luciferase activity. (c) The downregulation of circHIPK3 significantly upregulated the expression of miR-107. (d) Overexpression of miR-107 suppressed circHIPK3 expression. ^∗^*p* < 0.05, ^∗∗^*p* < 0.01, and ^∗∗∗^*p* < 0.001.

The bioinformatics analyses showed miR-107 was a potential binding miRNA for circHIPK3. We predicted the downstream target genes by bioinformatics tools (RegRNA) [20], and the prediction websites were http://regrna2.mbc.nctu.edu.tw/. The wild-type and mutant circHIPK3 luciferase reporter plasmids were constructed. Dual-luciferase reporter assay indicated cotransfection of miR-107, and WT-circHIPK3 reduced relative luciferase activity, while cotransfection of miR-107 and Mut-circHIPK3 did not result in the decrease of luciferase activity. These data confirmed that miR-107 was a direct target for circHIPK3 (Figures 3(a) and 3(b)). In order to explore the relationship between circHIPK3 and miR-107, we transfected miR-107 mimics or si-circHIPK3 into H1299 and A549 cells. The downregulation of circHIPK3 significantly upregulated the expression of miR-107 (Figure 3(c)), and the overexpression of miR-107 suppressed circHIPK3 expression (Figure 3(d)).

### 3.3. circHIPK3 Knockdown Suppressed H1299 and A549 Cell Proliferation through miR-107

**Figure 4:**
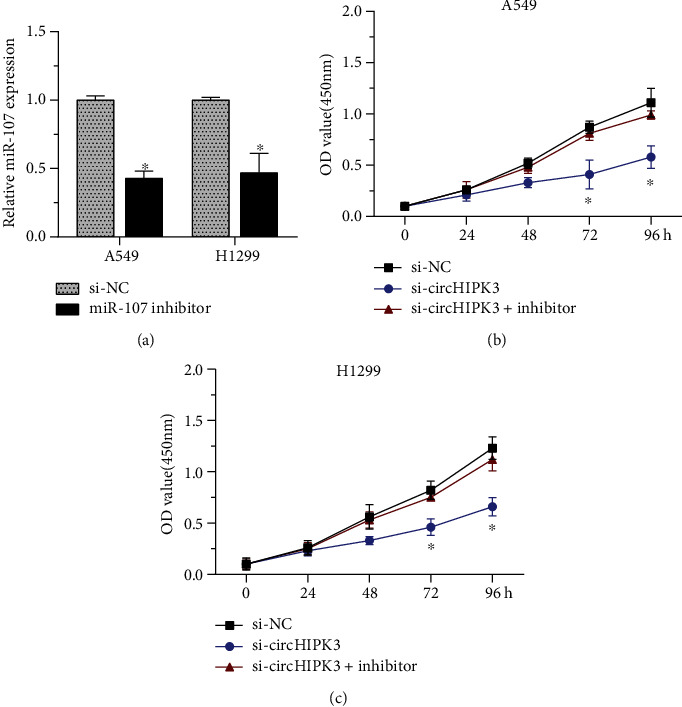
circHIPK3 knockdown suppressed H1299 and A549 cell proliferation through miR-107. (a) The miR-107 expression level of H1299 and A549 cells was significantly reduced after transfection with miR-107 inhibitor. (b, c) Downregulation of circHIPK3 suppressed the growth of A549 (b) and H1299 (c) cells. ^∗^*p* < 0.05, ^∗∗^*p* < 0.01, and ^∗∗∗^*p* < 0.001.

To determine whether miR-107 was involved in the effects of circHIPK3 knockdown on cell proliferation, H1299 and A549 cells were transfected with a miR-107 inhibitor that suppressed the expression of miR-107 (Figure 4(a)). The CCK-8 assay showed that the downregulation of circHIPK3 inhibited the proliferation of A549 and H1299 cells (Figures 4(b) and 4(c)). The downregulation of miR-107 reversed the suppressive effect, indicating that miR-107 was the downstream of circHIPK3. Our results proved miR-107 also played a key role in regulating tumorigenesis of NSCLC cell.

### 3.4. BDNF Was a Direct Target of miR-107

**Figure 5:**
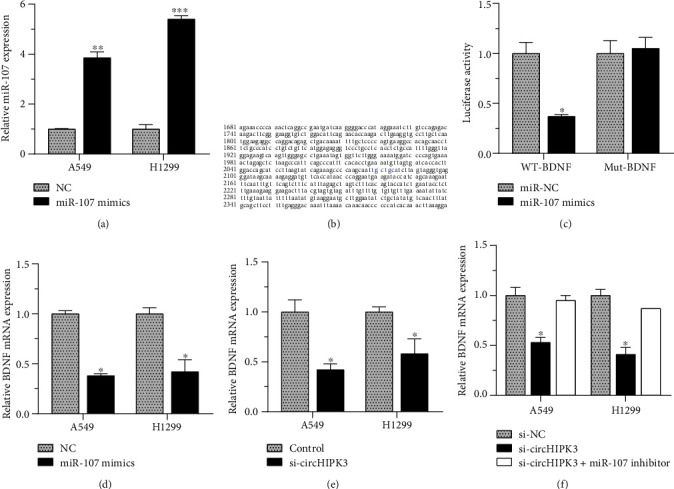
BDNF was a direct target of miR-107. (a) miR-107 expression increased after the transfection of miR-107 mimics in H1299 and A549 cells. (b, c) BDNF was a direct target of miR-107. (d) miR-107 overexpression remarkably inhibited the expression of BDNF. (e) circHIPK3 silencing suppressed BDNF mRNA levels. (f) miR-107 inhibitors had reversion function on the suppression caused by circHIPK3 silencing. ^∗^*p* < 0.05, ^∗∗^*p* < 0.01, and ^∗∗∗^*p* < 0.001.

BDNF plays a vital part in elevating the ability of tumor metastasis and proliferation. We explored the roles of BDNF in circHIPK3/miR-107-mediated tumor progression in NSCLC. Dual-luciferase reporter assay discovered cotransfection of miR-107, and BDNF 3′UTR reporter reduced dual-luciferase activity, while cotransfection of miR-107 and Mut-BDNF vectors proved to have no significant impact on relative luciferase activity. Our findings indicated BDNF was a direct target of miR-107 (Figures 5(b) and 5(c)). To further confirm the interaction between miR-107 and BDNF, the levels of BDNF mRNA in H1299 and A549 cells were detected after the upregulation of miR-107. The overexpression of miR-107 remarkably suppressed the expression of BDNF mRNA (Figure 5(d)), revealing that BDNF was a downstream regulator of miR-107. To confirm that BDNF was a target of circHIPK3, we detected BDNF mRNA levels after circHIPK3 knockdown, and the results showed circHIPK3 silencing suppressed BDNF mRNA levels (Figure 5(e)), and this suppression could be reversed by miR-107 inhibitors (Figure 5(f)).

**Figure 6:**
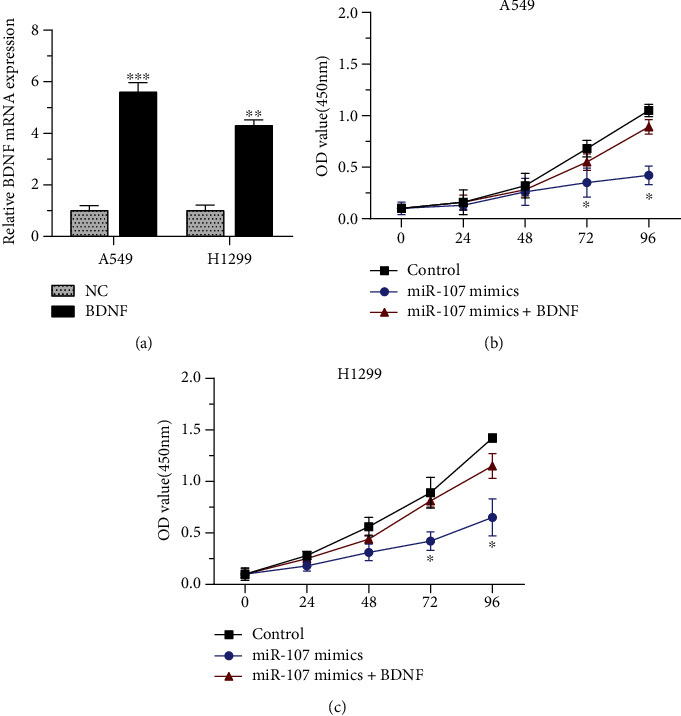
BDNF reversed the inhibition of miR-107 overexpression. (a) BDNF overexpression vector increased the BDNF mRNA expression. (b) The overexpression of BDNF had reversion function on the inhibition of miR-107 mimics on the proliferation of H1299 and A549 cells. ^∗^*p* < 0.05, ^∗∗^*p* < 0.01, and ^∗∗∗^*p* < 0.001.

Next, we constructed a BDNF overexpression vector (Figure 6(a)) and transfected it into NSCLC cells. The overexpression of BDNF reversed the inhibition of miR-107 mimics on the proliferation of H1299 and A549 cells, as proved in the CCK-8 assay (Figure 6(c)).

## 4. Discussion

Previous investigations demonstrated that circRNAs acted as a key regulator in the progression and development of cancers [11, 18]. According to the findings, we summarized that circHIPK3 played an important role in the proliferation and metastasis of NSCLC cells. In this study, we reported circHIPK3 was upregulated in NSCLC cells. Knockdown of circHIPK3 obviously reduced NSCLC cell proliferation, migration, and invasion. Our results showed the levels of miR-107 and BDNF expression were also regulated by circHIPK3 knockdown. This suggested that circHIPK3 promoted NSCLC development via the miR-107/BDNF axis.

It is evident that the circRNAs have a crucial part in the regulation of gene expression. circRNAs negatively regulate the miRNA level by direct binding, thus working as miRNA sponges in gene translation regulatory networks [21]. Our results showed that circHIPK3 bound to miR-107, which was further confirmed by the dual-luciferase reporter assay. Previous studies suggested that the expression of miR-107 was associated with the development of the disease. In Alzheimer's disease, miR-107 might target *β*-site amyloid precursor protein-cleaving enzyme 1 [22]. Chen et al. proved that miR-107 directly interacted with miRNA let-7 to negatively regulate the tumor suppressor, thereby promoting breast cancer cell growth [23]. Lee et al. found that the expression of cyclin-dependent kinase 6 in pancreatic cancer lines MiaPACA-2 and PANC-1 was regulated by the downregulation of miR-107 [24]. In this study, the knockdown of circHIPK3 upregulated miR-107 expression. However, the expression of downregulation of miR-107 reversed the circHIPK3-silencing-induced inhibition of the progression of NSCLC cells; accordingly, miR-107 upregulation suppressed NSCLC cell proliferation and metastasis.

BDNF exists in the adult central nervous system and human platelets [25]. At first, it was known BDNF had a crucial role in the development and function of the nervous system and cardiovascular system [26]. A series of studies indicated BDNF was associated with the development of many diseases. These findings showed that BDNF was aberrantly expressed in multiple human cancers, including bladder cancer and colorectal cancer [27, 28]. Wang et al. demonstrated that BDNF promoted thyroid cancer cell growth via regulating downstream signal pathway PI3K/AKT [29]. Xia et al. revealed miR-107 upregulation could suppress the progression of NSCLC through the downregulation of BDNF [30]. It was confirmed by our experimental results that miR-107 interacted with BDNF. In this study, mechanism assays suggested after circHIPK3 knockdown, miR-107 was released and the miR-107 downstream target genes, such as BDNF, were downregulated. Meanwhile, when we knocked down the expression of circHIPK3, the progression of NSCLC cells was suppressed, accompanied by the reduced expression of the miR-107 downstream targeted genes BDNF.

There are a number of factors, including the occurrence and development of NSCLC, including smoking, air pollution, and genetic [31, 32]. Apparently, a single study precludes a complete understanding of the etiology of NSCLC. Therefore, more basic studies are needed in the future to get more information about NSCLC.

In short, our results uncovered circHIPK3 new mechanism in regulating NSCLC proliferation and metastasis via acting as a miR-107 sponge. We highlighted circHIPK3/miR-107/BDNF as a novel tumor screening biomarker for NSCLC. Thus, we hoped that our findings would help to the screening and treatment of NSCLC. This regulatory mechanism could help us to explore the transcriptional regulation level in NSCLC and could be also explored in other diseases. This research result provided an important supplement to the regulatory study of the ceRNA regulation network.

## Data Availability

The datasets used and/or analyzed during the current study are available from the corresponding author on reasonable request.

## References

[1] Bray F., Ferlay J., Soerjomataram I., Siegel R. L., Torre L. A., Jemal A. (2018). Global cancer statistics 2018: GLOBOCAN estimates of incidence and mortality worldwide for 36 cancers in 185 countries. *CA: A Cancer Journal for Clinicians.*.

[2] Gu S., Jin L., Zhang F., Sarnow P., Kay M. A. (2009). Biological basis for restriction of microRNA targets to the 3′ untranslated region in mammalian mRNAs. *Nature Structural & Molecular Biology.*.

[3] Oser M. G., Niederst M. J., Sequist L. V., Engelman J. A. (2015). Transformation from non-small-cell lung cancer to small-cell lung cancer: molecular drivers and cells of origin. *The Lancet Oncology.*.

[4] Lv Y.-L., Yuan D.-M., Wang K. (2011). Diagnostic performance of integrated positron emission tomography/computed tomography for mediastinal lymph node staging in non-small cell lung cancer: a bivariate systematic review and meta-analysis. *Journal of Thoracic Oncology*.

[5] He J., Hu Y., Hu M., Li B. (2015). Development of PD-1/PD-L1 Pathway in Tumor Immune Microenvironment and Treatment for Non-Small Cell Lung Cancer. *Scientific Reports*.

[6] Yang L., Fu J., Zhou Y. (2018). Circular RNAs and Their Emerging Roles in Immune Regulation. *Frontiers in Immunology*.

[7] Shang Q., Yang Z., Jia R., Ge S. (2019). The novel roles of circRNAs in human cancer. *Molecular Cancer*.

[8] Zhou R., Wu Y., Wang W. (2018). Circular RNAs (circRNAs) in cancer. *Cancer Letters.*.

[9] Cao Q., Shi Y., Wang X. (2019). Circular METRN RNA hsa_circ_0037251 promotes glioma progression by sponging miR-1229-3p and regulating mTOR expression. *Scientific Reports*.

[10] Du W. W., Yang W., Liu E., Yang Z., Dhaliwal P., Yang B. B. (2016). Foxo 3 circular RNA retards cell cycle progression via forming ternary complexes with p 21 and CDK2. *Nucleic Acids Research.*.

[11] Zhang H.-d., Jiang L.-h., Sun D.-w., Hou J.-c., Ji Z.-l. (2018). CircRNA: a novel type of biomarker for cancer. *Breast Cancer*.

[12] Han X.-T., Jiang J. Q., Li M. Z., Cong Q. M. (2020). Circular RNA circ-ABCB10 promotes the proliferation and invasion of thyroid cancer by targeting KLF6. *European Review for Medical and Pharmacological Sciences*.

[13] Yang C., Yuan W., Yang X. (2018). Circular RNA circ-ITCH inhibits bladder cancer progression by sponging miR-17/miR-224 and regulating p21, PTEN expression. *Molecular Cancer*.

[14] Chen X., Mao R., Su W. (2020). Circular RNAcircHIPK3modulates autophagy viaMIR124-3p-STAT3-PRKAA/AMPK*α* signaling in STK11 mutant lung cancer. *Autophagy*.

[15] Lu H., Han X., Ren J., Ren K., Li Z., Sun Z. (2020). Circular RNA HIPK3 induces cell proliferation and inhibits apoptosis in non-small cell lung cancer through sponging miR-149. *Cancer Biology & Therapy*.

[16] Wang K., Liu C.-Y., Zhou L.-Y. (2015). APF lncRNA regulates autophagy and myocardial infarction by targeting miR-188-3p. *Nature Communications*.

[17] Zhou B., Yu J.-W. (2017). A novel identified circular RNA, circRNA_010567, promotes myocardial fibrosis via suppressing miR-141 by targeting TGF-*β*1. *Biochemical and Biophysical Research Communications.*.

[18] Meng S., Zhou H., Feng Z. (2017). CircRNA: functions and properties of a novel potential biomarker for cancer. *Molecular Cancer.*.

[19] Bai J., Zhu X., Ma J., Wang W. (2015). miR-205 regulates A549 cells proliferation by targeting PTEN. *International Journal of Clinical and Experimental Pathology*.

[20] Chang T.-H., Huang H.-Y., Hsu J. B.-K., Weng S.-L., Horng J.-T., Huang H.-D. (2013). An enhanced computational platform for investigating the roles of regulatory RNA and for identifying functional RNA motifs. *BMC Bioinformatics*.

[21] Cheng J., Zhuo H., Xu M. (2018). Regulatory network of circRNA–miRNA–mRNA contributes to the histological classification and disease progression in gastric cancer. *Journal of Translational Medicine*.

[22] Wang W.-X., Rajeev B. W., Stromberg A. J. (2008). The Expression of MicroRNA miR-107 Decreases Early in Alzheimer's Disease and May Accelerate Disease Progression through Regulation of -Site Amyloid Precursor Protein-Cleaving Enzyme 1. *Journal of Neuroscience*.

[23] Chen P.-S., Su J.-L., Cha S.-T. (2011). miR-107 promotes tumor progression by targeting the let-7 microRNA in mice and humans. *The Journal of Clinical Investigation.*.

[24] Lee K. H., Lotterman C., Karikari C. (2009). Epigenetic silencing of microRNA miR-107 regulates cyclin-dependent kinase 6 expression in pancreatic cancer. *Pancreatology*.

[25] Yamamoto H., Gurney M. (1990). Human platelets contain brain-derived neurotrophic factor. *The Journal of Neuroscience.*.

[26] Pius-Sadowska E., Machaliński B. (2017). BDNF – a key player in cardiovascular system. *Journal of Molecular and Cellular Cardiology.*.

[27] Zhi H., Lian J. (2019). LncRNA BDNF-AS suppresses colorectal cancer cell proliferation and migration by epigenetically repressing GSK-3*β* expression. *Cell Biochemistry and Function.*.

[28] Lai P. C., Chiu T. H., Huang Y. T. (2010). Overexpression of BDNF and TrkB in human bladder cancer specimens. *Oncology Reports*.

[29] Wang P., Meng X., Huang Y. (2017). MicroRNA-497 inhibits thyroid cancer tumor growth and invasion by suppressing BDNF. *Oncotarget*.

[30] Xia H., Li Y., Lv X. (2016). MicroRNA-107 inhibits tumor growth and metastasis by targeting the BDNF-mediated PI3K/AKT pathway in human non-small lung cancer. *International Journal of Oncology*.

[31] Jiang C., Fang X., Zhang H. (2017). AMD3100 combined with triptolide inhibit proliferation, invasion and metastasis and induce apoptosis of human U2OS osteosarcoma cells. *Biomedicine & Pharmacotherapy.*.

[32] Akhtar N., Bansal J. G. (2017). Risk factors of lung cancer in nonsmoker. *Current Problems in Cancer.*.

